# Single Shot Adductor Canal Block for Postoperative Analgesia of Pediatric Patellar Dislocation Surgery: A Case-Series Report

**DOI:** 10.1097/MD.0000000000002217

**Published:** 2015-12-07

**Authors:** Jia-Yu Chen, Na Li, Yong-Qing Xu

**Affiliations:** From the Department of Orthopedics (J-YC, Y-QX) and Department of Anesthesiology (NL), Kunming General Hospital of Chengdu Military Region, Kunming, Yunnan, China.

## Abstract

Postoperative analgesia for the knee surgery in children can be challenging. Meanwhile acute pain management in pediatric patients is still often undertreated due to inadequate pain assessment or management.

We reported the ultrasound-guided single-injection adductor canal block (ACB) with 0.2% ropivacaine and dexmedetomidine (0.5 μg/kg) in addition in a series of 6 children. Patients’ age was range from 7 to 15 years old with right or left habitual patellar dislocation needing an open reduction and internal refixation. Pain assessments using Numeric Rating Scale scores on the operative limb were made preoperatively and at 12, 24, 36, and 48 h postoperatively at rest. Medication consumption was calculated as well. The possible complications, such as hemodynamic changes, nausea, vomiting, and dysesthesia, were also recorded at 12, 24, 36, and 48 h postoperatively at rest.

The pain scores were low, and analgesic medication consumption was minimal. Meanwhile, no adverse events were recorded in any of the subject.

Single-injection ACB might be an optimal analgesia strategy for patellar dislocation surgery in pediatric patients.

## INTRODUCTION

However, the knee surgery in children or teenager is not common,^1^ it belongs to the most painful procedures with a need for fast recovery to gain function and prevent complications such as thromboembolic incidents.^2^ Meanwhile acute pain management in pediatric patients is still often undertreated due to inadequate pain assessment or management.^3,4^ Postoperative pain control for such patients is challenge to orthopedists. Pediatric regional anesthesia seem to be safety and an ideal method to minimize postoperative pain evidently.^5^ The adductor canal block (ACB) is a relatively new block providing analgesia for knee surgery, which not only blocks the largest sensory branch of the femoral nerve but also results in less reduction of quadriceps muscle strength, compared with the femoral nerve block (FNB) in adult patients.^6,7^ Dexmedetomidine (DEX) as an additive to local anesthetic provides a significantly longer postoperative analgesia with comparable adverse effects and hemodynamic changes, when compared with local anesthetics alone in pediatric regional anesthesia.^8^ We report 6 cases in which we successfully performed ultrasound-guided a single dose of local anesthetic with DEX in the adductor canal releasing postoperative pain of patellar dislocation surgery in children. Committee of Kunming General Hospital of Chengdu Military Command approved this study (2015031).

## METHODS

From April 2015 to July 2015, 6 pediatric patients who had undergone elective, unilateral open reduction and internal refixation, received postoperative analgesia using ultrasound-guided a single dose of local anesthetic with DEX in the adductor canal. This study was approved by Committee of Kunming General Hospital of Chengdu Military Command (2015031).

After induction of general anesthesia, patient received a single-injection ACB on operative leg with 0.2% ropivacaine and dexmedetomidine (0.5 μg/kg) in addition (10 mL). The blocks were performed during real-time ultrasonography using a color Doppler ultrasound machine (IU22, Philips, Amsterdam, The Netherlands) with a high-frequency linear transducer as previous described.^6^ The adductor canal was identified in short-axis view approximately at the mid-thigh level. A 0.5 × 35 mm needle was inserted in plane with the transducer with the tip of the needle located anterior to the superficial femoral artery, deep to the sartorius muscle, between the vastus medialis of the quadriceps muscle and the adductor longus muscle. All blocks were performed during real-time ultrasonography by J-YC and NL.

Pain assessments using Numeric Rating Scale (NRS) scores on the operative limb were made preoperatively and at 12, 24, 36, and 48 h postoperatively at rest. Medication consumption (MC) was calculated as well. The possible complications, such as hemodynamic changes, nausea, vomiting, and dysesthesia were also recorded at 12, 24, 36, and 48 h postoperatively at rest by J-YC.

### Cases Description and Results

Subject 1 is a 7-year-old girl, 15 kg with right habitual patellar dislocation needing an open reduction and internal refixation. After induction of general anesthesia, patient received a single-injection ACB on right leg with 0.2% ropivacaine and dexmedetomidine 7.5 μg (0.5 μg/kg) in addition (10 mL).

Subject 2 is an 8-year-old girl, 22 kg with left habitual patellar dislocation needing an open reduction and internal refixation. After induction of general anesthesia, patient received a single-injection ACB on left leg with 0.2% ropivacaine and dexmedetomidine 11 μg (0.5 μg/kg) in addition (10 mL).

Subject 3 is an 8-year-old boy, 21.5 kg with right habitual patellar dislocation needing an open reduction and internal refixation. After induction of general anesthesia, patient received a single-injection ACB on right leg with 0.2% ropivacaine and dexmedetomidine 10.75 μg (0.5 μg/kg) in addition (10 mL).

Subject 4 is an 11-year-old girl, 36 kg with left habitual patellar dislocation needing an open reduction and internal refixation. After induction of general anesthesia, patient received a single-injection ACB on left leg with 0.2% ropivacaine and dexmedetomidine 18 μg (0.5 μg/kg) in addition (10 mL).

Subject 5 is a 12-year-old boy, 50 kg with right habitual patellar dislocation needing an open reduction and internal refixation. After induction of general anesthesia, patient received a single-injection ACB on right leg with 0.2% ropivacaine and dexmedetomidine 25 μg (0.5 μg/kg) in addition (10 mL).

Subject 6 is a 15-year-old girl, 52 kg with left habitual patellar dislocation needing an open reduction and internal refixation. After induction of general anesthesia, patient received a single-injection ACB on left leg with 0.2% ropivacaine and dexmedetomidine 26 μg (0.5 μg/kg) in addition (10 mL).

Pain scores and MC are presented in Table 1. There were no hemodynamic changes in all 6 subjects. No adverse events were recorded in any of the subject.

**TABLE 1:**
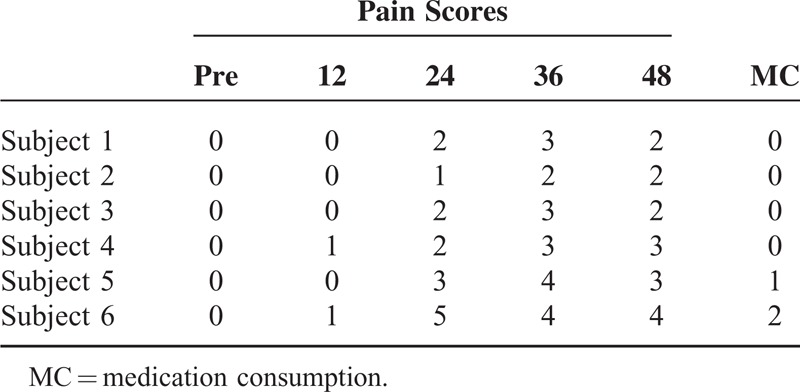
Data of Pain Scores and Medication Consumption

## DISCUSSION

Peripheral nerve block thought to be effective and safe method for postoperative analgesia and has attained wide use even in pediatric patients. Lee et al^9^ reported that peripheral nerve block found to be superior to systemic opioids for postoperative pain relief. Another study showed the effect of peripheral nerve block was similar to that caused by epidural analgesia.^10^ The complications of the systemic opioids and epidural analgesia, such as hypotension, respiratory depression, nausea/vomiting, and urinary retention, were not exclusive to the peripheral nerve block.^9–12^

The patellar surgery is associated with intense postoperative pain in pediatric patients. The challenge of treating postoperative pain after patellar surgery in pediatric patients is using easier analgesia method to provide optimal analgesia with preserved muscle function and minimal side effect. Single-shot analgesia might be better management than continuous catheter analgesia after surgery in pediatric patients. The ACB is a relatively new block. The largest sensory branch of the femoral nerve (the saphenous nerve), the medial femoral cutaneous nerve and probably articular branches of the obturator nerve and the nerve to the vastus medialis, a motor nerve but also the second largest sensory branch of the femoral nerve, are traversing the adductor canal.^6^ The most important finding was the ACB preserved the ability to ambulate better than the FNB.^7^ The study showed that the ACB only reduced the quadriceps muscle strength by 8% compared with baseline. In comparison, the FNB reduced the quadriceps strength by 49% compared with baseline.^7^ To our knowledge, there have been no published reports of management postoperative pain with ACB in pediatric patients.

Dexmedetomidine as a perineural adjuvant can prolong the durations of both sensory and motor block.^13–17^ However, there are presently insufficient safety data to support the use of perineural dexmedetomidine in the clinical setting.^13^ In pediatric patients, adjunct use of dexmedetomidine is associated with prolonged block duration following ilioinguinal/iliohypogastric nerve block. Furthermore, dexmedetomidine significantly reduces early break-through pain in the PACU.^18^ A meta-analysis shows that dexmedetomidine as an additive to local anesthetic provides a significantly longer postoperative analgesia with comparable adverse effects and hemodynamic changes, when compared to local anesthetics alone.^8^ In this study, we use 0.5 μg/kg dexmedetomidine in addition. Almost all subjects showed satisfactory score except subject 6 at 12 and 24 h postoperatively at rest. Even more, subject 1 to 4 showed low NRS scores at 36 and 48 h postoperatively at rest and did not require the supplemental analgesia within 48 h postoperatively. However, we do not establish the control group which did not add dexmedetomidine, the 6 subjects showed acceptable NRS scores and no adverse events during 48 h after surgery. Subjects 5 and 6 showed comparatively high scores at 24 and 36 h after surgery. Andersen et al took a study in the spread of injectate during saphenous nerve block at the adductor canal on adult cadavers. They recommend 15 ml could be an optimal dosage to spread throughout the adductor canal and beyond both proximally and distally.^19^ So we presume that might be a low volume reason for over 50 kg patients which resulted in insufficient pain management.

A retrospective study hints that single-injection ACB offered similar pain control and earlier discharge compared to continuous femoral nerve catheter in patients undergoing total knee arthroplasty (TKA).^20^ Single-injection adductor canal blockades may be superior for postoperative ambulation, knee flexion, and providing similar pain control and analgesics requirements. Our limitation is that this study is just a case-series report. Whether dexmedetomidine could prolong the durations of postoperative analgesia using single-injection ACB and the safety dosage need further clinical trials study.

## CONCLUSIONS

We successfully performed ultrasound-guided single shot of local anesthetic with DEX in the adductor canal releasing postoperative pain of patellar dislocation surgery in 6 pediatric patients. The patients and their parents reported comparatively satisfactory scores. Further study is needed to determine more widespread application of this technique.
